# Timing and extent of Peri-Implant bone loss for dental implant removal: A retrospective Cross-Sectional analysis

**DOI:** 10.1007/s00784-026-06774-2

**Published:** 2026-03-07

**Authors:** Tim Halstenbach, Wiebke Semper-Hogg, Fabian Cieplik, Erik Würflein, Florian Kernen, Marc Metzger, Rainer Schmelzeisen

**Affiliations:** 1 Department of Oral- and Craniomaxillofacial Surgery, Medical Center, Faculty of Medicine, University of Freiburg, Hugstetter Straße 55, 79106 Freiburg, Germany; 2 Department of Operative Dentistry and Periodontology, Medical Center, Faculty of Medicine, University of Freiburg, Hugstetter Straße 55, 79106 Freiburg, Germany

**Keywords:** Dental implantology, Peri-implantitis, Implant loss

## Abstract

**Objectives:**

The occurrence of dental implant loss has not been systematically investigated. This study assessed the extent of radiological crestal bone loss at time of dental implant removal and the timing of dental implant removal.

**Materials and methods:**

Patients transferred to the Department of Oral- and Craniomaxillofacial Surgery of the University clinic of Freiburg, Germany, between 2014 and 2024 for dental implant removal were retrospectively analyzed. Radiological bone loss was determined using panoramic and intraoral radiographs. Further data included patient demographics, implant characteristics, bone defect morphology, and specific reasons for removal.

**Results:**

738 implant removals in 462 patients were included. Peri-implant inflammation accounted for 71% of implant removals, with 83% of late implant losses attributed to this cause. The mean radiological bone loss at time of implant removal was 51% (SD: 28%) and was significantly higher in the mandible than in the maxilla (*p* < 0.001). Most implants were lost three years after placement. At later time points, less implants were removed. Average bone loss levels at the time of implant removal increased over time, ranging from 36% in the first three years to 60% in years 8 to 10 (*p* = 0.002). Complete loss of osseointegration was observed more frequently over time.

**Conclusion:**

This study provides the first large-scale systematic assessment of dental implant loss. Peri-implant bone loss was confirmed as the leading cause of dental implant failure. Within this monocentric clinical setting, dental implant removal was closely associated with a radiographic bone loss threshold of around 50%, underscoring its potential value for clinical decision-making in advanced peri-implant disease.

**Clinical relevance:**

A radiographic bone loss level of approximately 50 % may serve as a pragmatic threshold to escalate from salvage concepts towards the decision of implant removal in advanced peri-implant disease. The high proportion of implant removals occurring in the early years after placement highlights the importance of early clinical surveillance.

**Supplementary Information:**

The online version contains supplementary material available at 10.1007/s00784-026-06774-2.

## Introduction

Dental implants are widely regarded as reliable options for the rehabilitation of the compromised jaw. Advances in osseointegration and peri-implant hard and soft tissue management have contributed to survival rates exceeding 90% over 20 years [1–3], enabling predictable and long-term treatment outcomes. However, with increasing use of dental implants [4] failure or loss of implants becomes a significant clinical concern.

Implant loss is categorized into early and late failures [5, 6]. Early implant failure occurs during osseointegration, prior to prosthetic loading, due to infections or inflammatory and non-inflammatory factors. Meta-analyses indicate that early implant loss affects approximately 1.5% of placed implants [5, 7]. Some authors have postulated that early implant loss should be seen as the unfavorable outcome of a foreign body reaction (FBR) [8–10]. Here, soft tissue encapsulation occurs leading to implant loosening and implant loss [11, 12]. Although immunological mechanisms contributing to this process have been assumed, robust evidence to confirm that early implant loss is primarily a manifestation of a foreign body reaction remains lacking [9, 10, 13].

Late implant loss, affecting long-term stability, is mainly driven by biological and biomechanical factors, with peri-implantitis being the leading cause [3, 5]. Peri-implantitis is an inflammatory disease of the implant surrounding tissue, characterized by progressive loss of crestal bone [14].

Procedures for regaining the lost attachment by guided bone regeneration have been described, but their efficacy is constrained by defect size and morphology and remains underexplored in larger cohorts [15, 16]. Consequently, most treatments focus on reducing inflammation rather than fully resolving peri-implantitis or restoring osseous integration, making implant removal a frequent outcome [17, 18].

Despite extensive research on peri-implant diseases and therapeutic interventions, limited data exist on how and when peri-implantitis leads to implant loss. Existing studies focus on prevalence of peri-implantitis or risk factors, while dental implant loss has not been systematically investigated in regard to timing and associated bone loss. Understanding these temporal and morphological trends is essential for refining diagnostic thresholds, optimizing intervention timing, and developing evidence-based guidelines to prevent progression to irreversible implant failure. Therefore, this retrospective cross-sectional study aimed to analyze the timepoints of implant loss and to assess the associated crestal bone loss and defect morphology at the time of removal.

## Materials and methods

This retrospective, cross-sectional, monocentric study was approved by the ethics committee of the University of Freiburg, Germany (Vote: 23-1166_1-S1-retro). Records of patients transferred to the Department of Oral- and Maxillofacial Surgery of the Medical Center of the University of Freiburg, Germany, for dental implant removal between 2014 and 2024 were reviewed. All available patients were included. Because the cohort comprised implants referred for removal, this study characterizes terminal implant conditions and does not allow comparison with surviving implants or causal inference regarding risk factors or failure.

### Inclusion criteria

The following inclusion criteria applied:

- Age: 18 years or older at time of implant removal.
- Availability of panoramic radiographs or intraoral radiographs of appropriate quality taken up to 6 months prior implant removal.
- Minimum of 1 dental implant to be removed.

If no recent radiograph of the implant was available or if radiographs were of inferior quality (e.g. mispositioning, low resolution, motion artefacts) or in case of incomplete depiction of the implant of interest, cases were excluded.

### Data acquisition

The following data was acquired:

- Gender of the patient.
- Age at time of implant placement and implant removal.
- Position (FDI) of the implant to be removed.
- Reason for the removal of the implant.
- Anamnestic factors, including previous radiation of the head and neck area, and intake of antiresorptive drugs.
- Date of implant removal.
- If available: Date of implant placement.
- If available: Implant manufacturer.
- Relative radiological bone loss at time of implant removal.
- Classification and grade of peri-implant bone defect, according to Monje et al. [19].

Implant removal before prosthetic loading was defined as early implant loss. Cases of late implant loss were subclassified as follows:

- Implant removal due to mechanical failure, including fracture of the implant or the abutment in irrecoverable extent.
- Implant removal in the course of osteonecrosis of the jaw, either in association with radiation (osteoradionecrosis) or with antiresorptive drugs (medication related osteonecrois of the jaw, MRONJ).
- Implant removal due to peri-implant inflammation, including peri-implantitis [20], patients reporting pain or discomfort associated with the implant, peri-implant suppuration, abscesses caused by dental implants, implant loosening.
- Implant removal due to aesthetic or prosthetic reasons.
- Implant removal due to implant displacement.

Since only insufficient clinical data on probing-depth and bleeding on probing was available, the term “peri-implant inflammation” was used as an umbrella category for advanced inflammatory clinical conditions leading to implant removal, including peri-implantitis according to the established case definitions as well as terminal manifestations such as suppuration/abscess formation, pain, and implant loosening.

### Evaluation of radiological bone loss

**Fig. 1:**
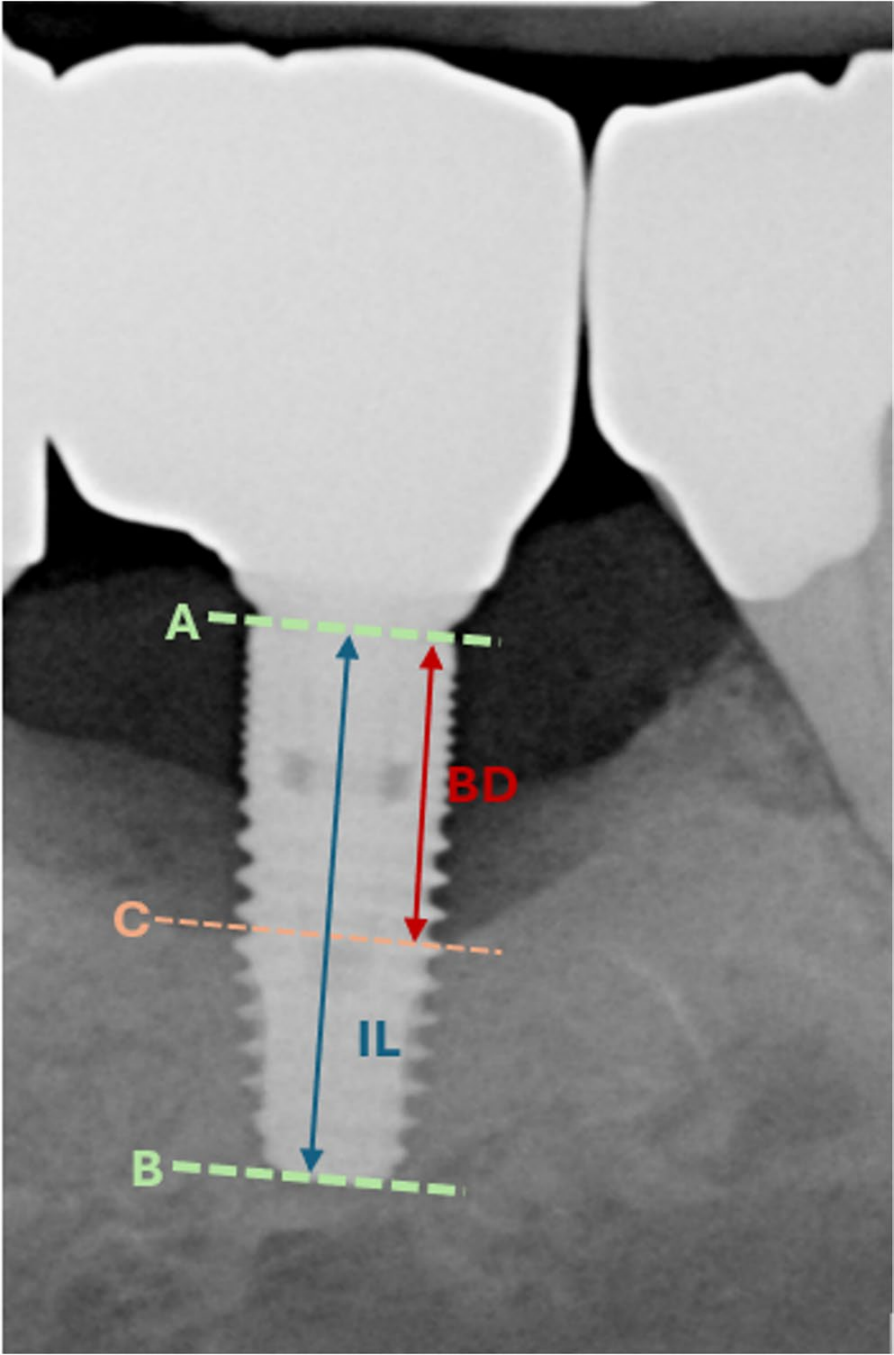
Bone Loss evaluation: For the assessment of relative bone loss (%BL) the ratio between the implant length (IL, distance A-B) and the bone defect (BD, distance A-C) was calculated. (**A**): most coronal point of the intraosseous part of the implant; (**B**) most apical part of the implant; (**C**) most apical part of the bone defect; (BD) Bone Defect; (IL) Implant Length

For bone level assessment, panoramic and intraoral radiographs were used. Previous studies have indicated, that both modalities allow for comparable accuracy for this task [21, 22]. Furthermore, the relative radiological bone loss was measured decreasing the effect of image distortion as observed in panoramic radiographs [23]. The relative radiological bone loss (Relative Bone loss, %BL) was calculated as described before [24], by measuring the implant length from the apex to the most coronal point of the intraosseous implant part (implant length, IL) and the most apical part of the osseous defect and the most coronal point of the intraosseous implant part (bone defect, BD) (see Fig. 1):

$$\:\%BL=\frac{BD}{IL}*100$$

If available, earlier radiographs were used to determine the baseline bone height. Radiological measurements were performed by an experienced clinician (TH) to minimize the risk of variability arising from operator-dependent factors. Previous studies have indicated good intra-rater agreement regarding the assessment of peri-implant bone heights [21, 25]. In cases of ambiguous bone levels a second measurement was performed by a second rater (WS-H) and results were averaged. DeepUnity Diagnost (V. 1.2.0.3, DH Healthcare GmbH, Germany) was used for analysis of radiographs.

### Evaluation of peri-implant bone defects

Peri-implant bone defects were radiologically classified using the system introduced by Monje et al. This classification was originally based on CBCT data, allowing for the differentiation of three primary defect types:

- **Class I**: Intraosseous defects.
- **Class II**: Supracrestal (horizontal) defects.
- **Class III**: Combined defects.

Classes I and III are further subdivided into three subgroups (a: buccal dehiscence; b: 2–3 walls defects; c: circumferential defects) [19]. As Monjes classification is based on three-dimensional imaging, assessment via two-dimensional modalities (intraoral and panoramic radiographs) does not allow to reliably distinguish between the subtypes [26, 27]. However, conventional radiographs can be used for the detection and differentiation of peri-implant bone defects with sufficient sensitivity, hence the present study relied on the use of intraoral and panoramic images for determining the primary defect classes (I-III) in mesial-distal orientation [28, 29].

Additionally, defect severity was graded based on vertical bone loss:

- **S (Slight)**: < 25% bone loss.
- **M (Moderate)**: 25–50% bone loss.
- **A (Advanced)**: > 50% bone loss.

This severity grading, as proposed by Monje et al., was applied in the current study.

### Statistical analysis

Statistical analysis was performed in Microsoft Excel^®^ and RStudio (v. 2024.09.1). For comparison between groups Analysis of Variance (ANOVA) was performed. Post hoc testing was performed with pairwise t-tests and Bonferroni-correction. The Pearson correlation coefficient and Mann-Kendall trend test were used to investigate changes over time.

## Results

In 462 patients (44% female, 56% male), a total number of 738 removed dental implants were included in the study. The mean age at time of implant removal was 66 years (+- 13 years). The cohort consisted of 403 implants (55%) in the maxilla and 335 (45%) implants in the mandible. The first molar was the most common implant position in the lower jaw to be removed, while in the maxilla, implants in the premolar or first molar regions were most frequently removed (Table 1).

**Table 1:** Cohort characteristics. 738 implants in 462 patients were observed. Table summarizes the main reasons for implant loss and the relative distribution of implant positions in the upper and lower jaw

*N* (implants)	738	
N (patients)	462	
Male	257	
Female	205	
Age		
Average age	65 y	
Median age	67 y	
Standard deviation	± 13 y	
IQR	58 y – 75 y	
Reasons for implant loss (%)		
Peri-implant inflammation	71.00%	
ARONJ	2.98%	
Osteoradionecrosis	0.27%	
Implant fracture	2.03%	
Mispositioning	1.22%	
Early implant loss	14.50%	
Other	7.99%	
Implant positions		
Jaw	Position	%
Maxilla	All	55%
Mandible	All	45%
Maxilla	8	0.27%
Maxilla	7	2.88%
Maxilla	6	11.78%
Maxilla	5	10.68%
Maxilla	4	11.51%
Maxilla	3	5.48%
Maxilla	2	7.81%
Maxilla	1	4.79%
Mandible	8	0.55%
Mandible	7	5.89%
Mandible	6	13.56%
Mandible	5	7.40%
Mandible	4	5.75%
Mandible	3	5.62%
Mandible	2	4.38%
Mandible	1	2.74%

Early implant loss occurred in 107 cases (14.5%). Late implant loss accounted for 85.5% with peri-implant inflammation as the leading cause (71% of cases of late implant loss). Other reasons for implant loss, such as implant fractures or osteonecrosis, accounted only for a small proportion of cases. In 8% of cases, the reason for implant removal remained unclear (Table 1).

**Fig. 2:**
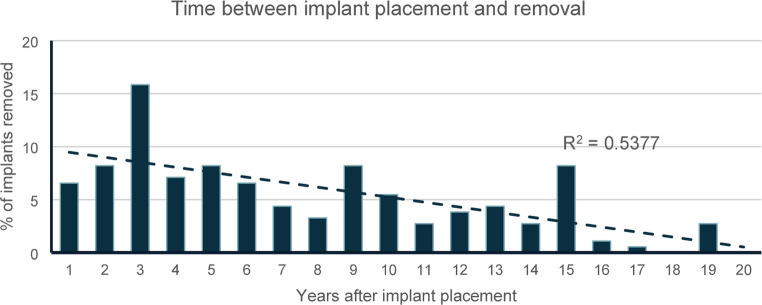
Time between implant placement and removal. The figure displays the time in years (1 to 20) between implant placement and implant removal. Y-axis indicates the proportion of implants that had to be removed in the respective year. Cases of implant loss before implant loading excluded, n = 183. Trendline indicated by dotted line

For further analysis of radiological bone loss, only implants lost due to inflammation were included (osteoradionecrosis and MRONJ excluded) (*n* = 524). The mean radiological bone loss level at time of implant removal was 51% (+- 28%, median: 49%, IQR: 32.43% − 67.05%). The mean bone loss level was higher in the mandible (mean 59% +- 31%, IQR: 37.64% – 81.78%) than in the maxilla (45% +−23%, IQR: 30.33% – 59.57%) (*p* < 0.001). Relative radiological bone loss at time of implant removal ranged from no radiological bone loss to complete loss of implant integration in both jaws. ANOVA with post-hoc t-tests revealed no significant differences in radiological bone loss related to implant position in either the maxilla or mandible (supplementary Fig. 1). Out of 291 implants with known timepoint of implant placement, 183 accounted for late implant loss. For these cases, the average time between implant placement and implant removal was 7.4 years (+- 4.9 years, median 6 years). Figure 2 illustrates the periods between implant placement and removal. 16% of the removed implants had been in situ for 3 years. While Pearson-correlation coefficient indicated moderate correlation between implant loss and time after implant placement (*r* = 0.54), Mann-Kendall trend test revealed statistical significance (τ = −0.645, *p* < 0.001).

**Fig. 3:**
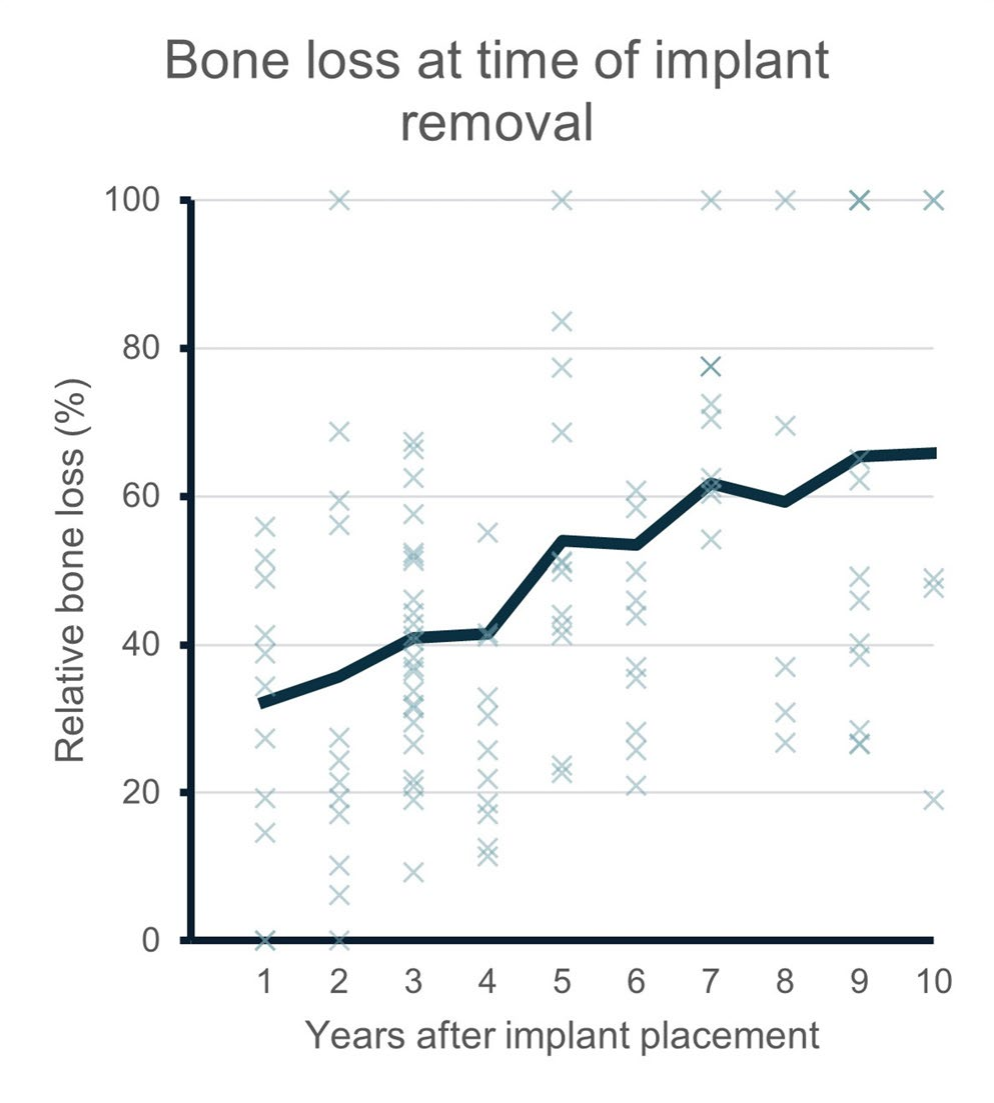
Moving average of bone loss at time of implant removal. Blue line indicates the average radiological bone loss (%) of +- one year for years 1 to 10 after implant placement. Radiological bone loss levels at the time of implant removal increased over time and the frequency of complete loss of osseointegration (100 % of radiological bone loss) was observed more frequently in later years

Implants removed after longer functional periods exhibited significantly higher radiographic bone loss levels at the time of removal compared to implants removed within the first three years (mean bone loss level: 36% (+- 21%, IQR: 21% − 51%) in years 1–3 and 60% (+- 30%, IQR: 34% − 100%) by years 8–10 (*p* = 0.002)) (Fig. 3). Total loss of osseointegration (100% of crestal bone loss) was observed in 18% of cases in the first three years and in 28% of cases in years 8 to 10. Most of the implants examined in this study were manufactured by Straumann (38%) and Camlog (21%). For 32% of all cases the manufacturer of the implant remained unknown. Implant manufacturers are listed in supplementary Table 1. The observed manufacturer distribution likely reflects regional and institutional usage patters at the referring centers, rather than manufacturer-specific susceptibility to implant failure.

**Fig. 4:**
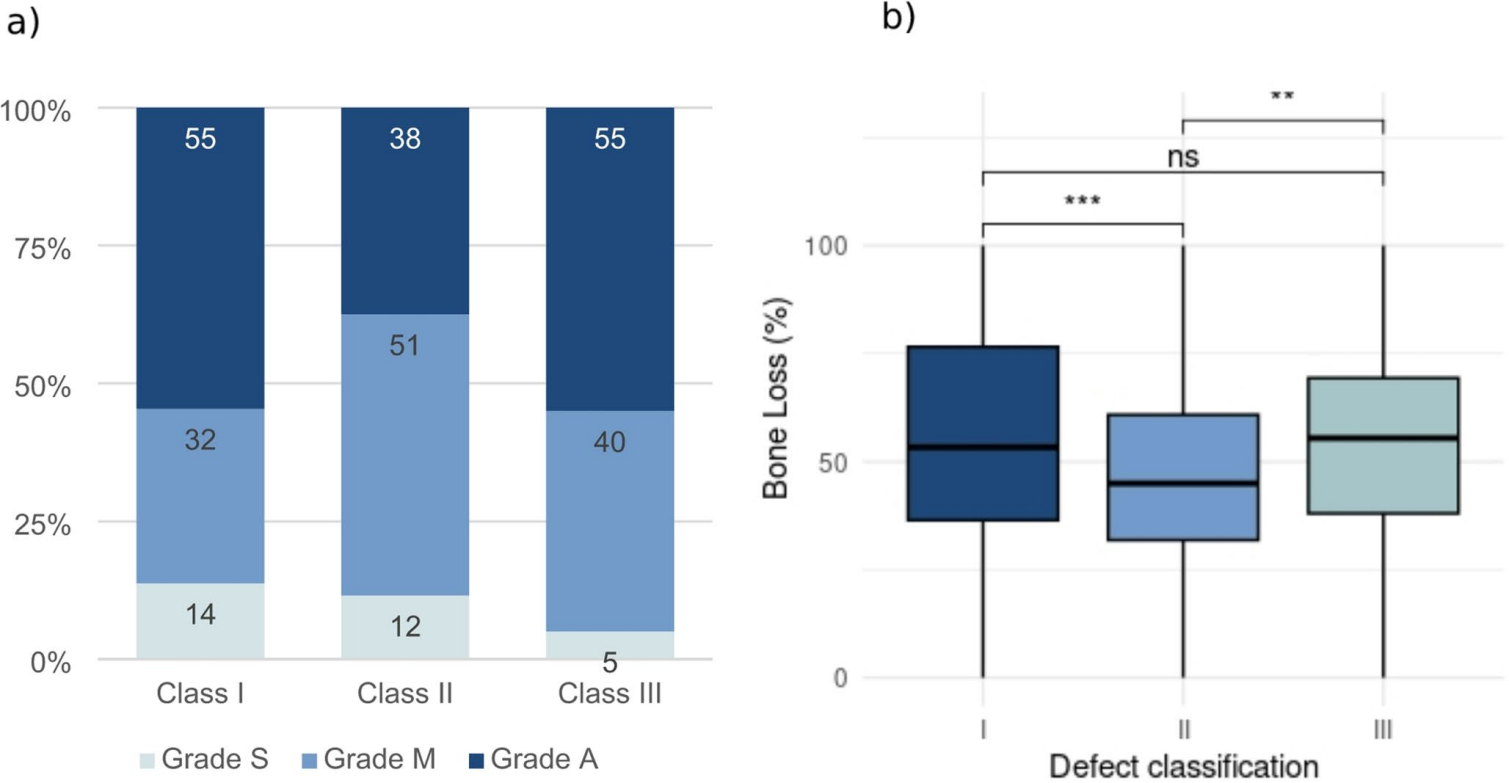
Morphology and severity of peri-implant bone defects classified according to Monje et al. (**a**) Grading: Grade A (Advanced, > 50 % radiological bone loss) was the most prevalent grade for classes I and III, while grade M (Moderate, 25-50 % radiological bone loss) was most prevalent for class II defects. (**b**) Bone loss in relation to defect classification: bone loss observed in class II defects was significantly lower compared to class I and III defects

**Fig. 5:**
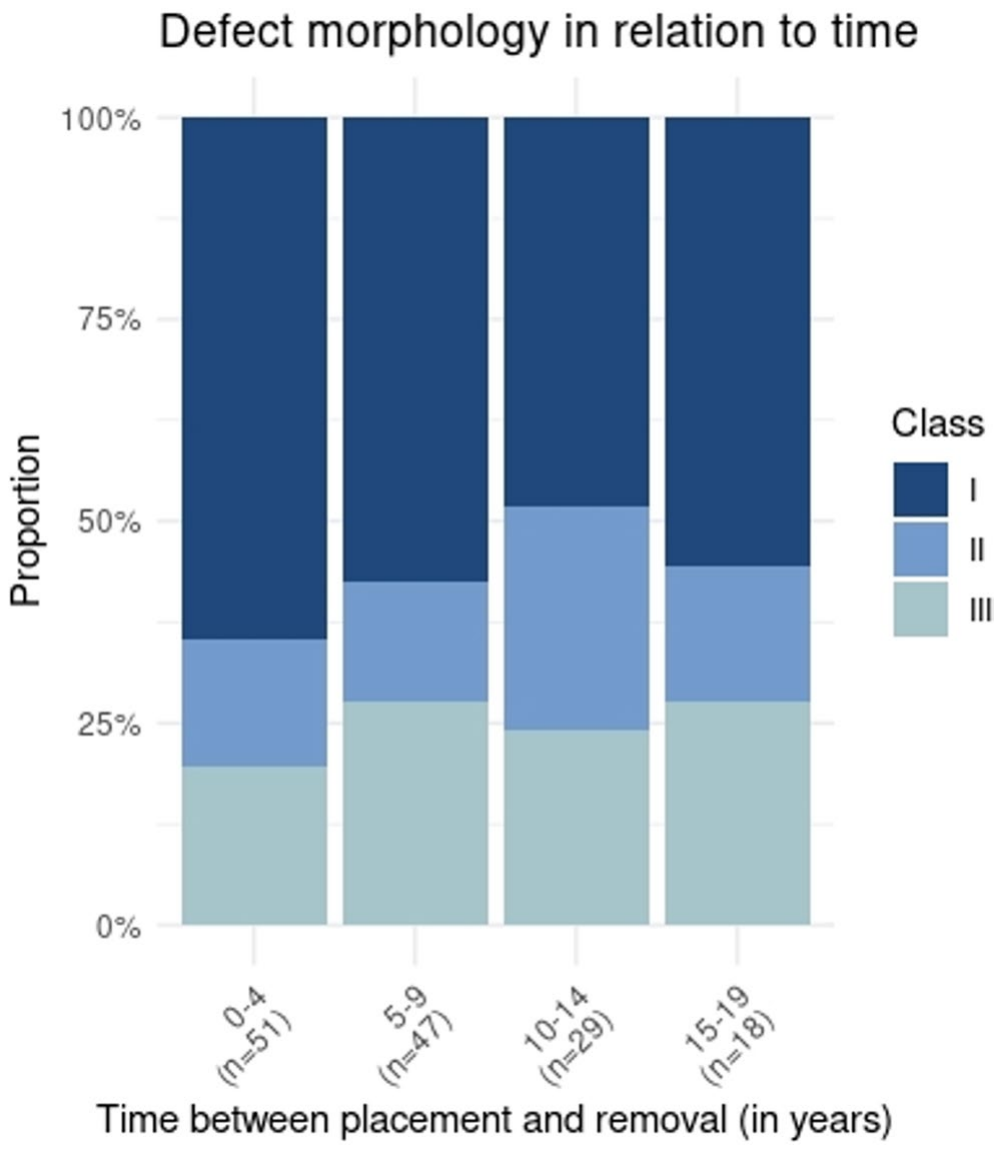
Defect morphology in relation to time. Peri-implant bone defects were classified according to Monje et al. 2019 in classes I-III. Figure depicts the proportion of each class in relation to time between implant placement and removal (in years)

The defect morphology was defined for all implants lost due to peri-implant inflammation. Defect class I accounted for 45.1%, class II for 21.6% and class III for 25% of cases. For 8.3% no radiological bone loss was observed at the time of implant removal and therefore the classification of Monje et al. did not apply. For 55% of defects classified as I or III, severity of bone loss was graded A (advanced) according to Monje et al., while grade M (moderate) accounted for 51% of class II defects. ANOVA (*p* = 0.004) and post-hoc t-tests with Bonferroni correction revealed significantly lower bone loss at time of implant removal for class II defects (average: 47% +−22%, median: 45%, IQR: 32% − 61%) compared to class I (average 57% +- 28%, median: 53%, IQR: 37% − 77%) (*p* = 0.0007) and class III (average: 55% +- 22%; median: 56%, IQR: 38% − 69%) (*p* = 0.0044). Differences between class I and III were not significant (*p* = 0.6051) (Fig. 4). Class I defects were most prevalent at all time points (Fig. 5). Differences in the proportion of the different defect classes over time were not significant (Pearson´s Chi-squared test: *p* = 0.7342).

## Discussion

This retrospective analysis of 738 removed dental implants identifies peri-implant bone loss as the leading cause of late implant failure. Most implants that were removed had been in function for only a few years with a peak at three years after implant placement, whereas fewer removals involved implants with longer functional durations. To our knowledge, this is the first systematic assessment of dental implant loss, demonstrating that the decision for implant removal is made at a mean of 50% of radiological bone loss.

The majority of losses following prosthetic loading occurred within the first years, then decreased progressively—a pattern aligning with the onset timeline of peri-implantitis, which predominantly manifests in the early post-loading phase. Notably, Derks et al. found that only 4% of peri-implantitis cases emerged beyond five years, reinforcing the temporal correlation between peri-implantitis and late implant failure [14]. Given that peri-implant inflammation accounted for 83% of late implant failures in the present study, it is reasonable to infer that implant loss timelines closely mirror the onset and progression of peri-implantitis.

Radiographic analysis indicated that implant failures in the first years after implant placement were typically associated with lower levels of bone loss, whereas advanced bone resorption and complete loss of osseointegration became increasingly prevalent in older implants. This aligns with previous findings highlighting the accelerated progression of peri-implantitis [14, 30]. Furthermore, declining adherence to maintenance therapy over time likely contributes to delayed diagnosis and treatment, resulting in more severe bone defects. This emphasizes the critical role of regular maintenance therapy in preventing disease progression and preserving implant stability [30–32]. Finally, clinicians may apply stricter explantation criteria to younger implants, whereas older implants may be retained longer and removed at more advanced stages.

Although current guidelines lack a universally defined bone loss threshold for implant removal, several studies have demonstrated a negative correlation between baseline bone loss and treatment outcomes for peri-implantitis [15–18]. Current reviews identified 50% of bone loss s an appropriate cutoff margin for the decision for implant removal, corresponding to the results of the present study [33, 34].

With respect to defect morphology, intraosseus bone defects (class I) were most prevalent at all time points, corroborating previous studies [19, 31, 35]. However, the proportion of horizontal (class II) defects was notably higher in this cohort (21%) than preiously reported (< 5%) [19]. This difference might result from the focus on terminal peri-implant inflammation cases with advanced defect severity. Class II defects present therapeutic challenges, typically permitting only resective treatment with poorer prognosis [16, 18]. The latter is reflected in the findings of this study, where class II defects were associated with significantly lower levels of bone loss when compared to class I and III defects.

The absence of Cone Beam Computed Tomography (CBCT) imaging is a relevant limitation, as buccal and lingual defect components cannot be reliably assessed with two-dimensional radiographs, which may impact the classification of peri-implant bone defects [26, 36, 37]. In this context, the high proportion of class I (intraosseous) defects observed in the present study may, at least in part, be attributable to the limited ability of two-dimensional imaging to detect buccal and lingual bone loss, potentially leading to an underestimation of combined defect configurations. Nevertheless, panoramic and intraoral radiographs reflect routine clinical diagnostics and thus the imaging basis on which therapeutic decisions are commonly made. Although CBCT offers enhanced spatial resolution, its utility is hampered by implant-induced artifacts and increased radiation exposure [36, 38, 39]. High-resolution dental MRI holds promise for future diagnostic use but is not yet standard in clinical practice [40]. Given these constraints, conventional radiographs remain the primary imaging modality for the assessment of peri-implant bone loss. Accordingly, our results represent the radiographic information available at the time of implant removal rather than three-dimensional defect quantification.

Peri-implantitis has frequently been described as being more prevalent in the maxilla than in the mandible [41–43]. This study reflects that trend, as 55% of rmoved implants were located in the maxilla. However, radiographic bone loss at the time of removal was significantly greater in the mandible. Differences in bone density and vascularization between jaws, with more porous, vascularized bone in the maxilla versus denser cortical bone in the mandible, may contribute to divergent disease progression and thresholds for clinical intervention [19, 42, 44, 45]. Additionally, greater bone density in the mandible may delay clinical symptoms such as implant loosening, leading to later removal at more advanced stages of bone loss [46]. These anatomical and physiological disparities suggest that clinical decisions regarding implant removal are influenced not solely by bone loss severity but also by functional concerns, patient-reported symptoms, and anatomical location.

Although peri-implant bone loss emerges as the dominant cause of late implant loss in this study, the decision to remove an implant remains multifactorial. Patient factors (e.g., systemic health and hygiene adherence), anatomical conditions, clinician experience, and socioeconomic considerations influence the decision to remove an implant, which underscores the necessity for a comprehensive, individualized approach to diagnosis and treatment planning [2, 47, 48].

An important limitation of the study is the selective retrospective assessment of removed implants, which inherently limits generalizability and precludes estimation of failure risk or survival analyses, as no denominator of placed and surviving implants was available. Furthermore, information about patient-level factors, such as smoking or a history of periodontitis, and clinical factors, such as probing depth or bleeding on probing, were not conclusively available, therefore limiting the ability to draw conclusions on the peri-implant inflammatory state and potential causal relations. The monocentric design of the study limits the generalizability of the findings. Center-specific factors, including surgical protocols, referral pathways, and maintenance strategies, likely influence the clinical decision to remove an implant. This potential bias may be further amplified by the fact that the cohort comprises implants referred for implant removal. Consequently, the observed mean radiographic bone loss should be interpreted with caution, as the present data do not allow conclusions regarding the therapeutic limits of peri-implantitis treatment [49–54].

To our knowledge, this is the first study to systematically correlate implant loss with radiographic bone loss across time. Future prospective studies utilizing three-dimensional imaging and incorporating clinical and patient-specific parameters are needed to validate these findings and refine criteria for timely and effective implant intervention.

## Conclusion

By systematically combining clinical data with radiographic bone loss and defect morphology at removal in a large cohort, our study adds novel evidence to the limited literature on terminal peri-implant disease. The study discloses the critical role of peri-implant inflammation in implant loss, with its early onset contributing to a high incidence of failures in the initial years post-placement. Radiological bone loss is a valuable, though not definitive, predictor of implant removal, particularly when exceeding 50% of attachment loss. The findings highlight the need for stringent maintenance protocols and early intervention to manage peri-implant disease and contribute to long-term success of oral rehabilitation.

## Supplementary Information

Below is the link to the electronic supplementary material.

Supplementary file 1 (DOCX 33.4 KB)

## Data Availability

No datasets were generated or analysed during the current study.
