# Supplementary material for: Timing and extent of Peri-Implant bone loss for dental implant removal: A retrospective Cross-Sectional analysis
Source: Clin Oral Investig. 2026 Mar 7;30(3):111. doi: 10.1007/s00784-026-06774-2 (PMC12967551; doi:10.1007/s00784-026-06774-2)
Supplement: Supplementary file 1 — (DOCX 33.4 KB) [file 784_2026_6774_MOESM1_ESM.docx]

Supplementary figure 1: Average bone loss at time of implant removal depending on implant position. Figure depicts the mean bone loss observed for tooth position 1 to 8 (FDI) in the maxilla (green) and the mandible (blue). Error bars indicate standard error. Dotted lines indicate average radiological bone loss for the respective jaw: upper jaw 45 %; lower jaw 59 %; p = 2.42E-7. ANOVA and subsequent post-hoc-testing showed no significant differences between implant position within one jaw.

| Manufacturer | % |
| --- | --- |
| Ankylos | 0.30 % |
| Astra | 1.07 % |
| Branemark | 1.22 % |
| Camlog | 21.16 % |
| Nobel | 0.91 % |
| SIC | 4.57 % |
| Straumann | 38.20 % |
| Trident | 0.15 % |
| Dentsply | 0.61 % |
| Zeramex | 0.15 % |
| Zimmer | 0.15 % |
| Unknown | 31.81 % |

Supplementary Table 1: Manufacturers of implants. The table displays the proportion of different implant manufacturers of all implants that were included in the study (n=738).
